# Why Wait? Neuroscience Is for Everyone!

**DOI:** 10.1523/ENEURO.0372-20.2022

**Published:** 2022-06-16

**Authors:** Norbert Myslinski

**Affiliations:** Department of Neural and Pain Sciences, University of Maryland, Baltimore, MD 21201

## Abstract

Neuroscience is not just for neuroscientists. It is for everyone, but it is absent from our high schools. High schools have a huge investment in STEM but do not include neuroscience, although neuroscience is more interesting and relevant to a person’s daily life than most other sciences. However, neuroscience opportunities are increasing for teenagers outside the standard curriculum.

## Significance Statement

The neuroscience community and the education community must provide more opportunities in neuroscience education for teenagers.

## Introduction

By its nature, neuroscience is one of the most fascinating and relevant sciences on earth. As a result, teenagers from around the world are inherently attracted to it. Unfortunately, neuroscience education opportunities in high schools are wanting. High school students ask their teachers for neuroscience courses and are told that it is taught in medical schools, that it is not a high school course. They are told that they have to wait for college. “Wait? Why wait? We want to learn about the human brain now,” I am told by students from around the world. They should not have to wait. Teachers and the educational community have a huge investment in STEM (Science, Technology, Engineering, and Mathematics) but do not include neuroscience in their curricula, although neuroscience is more interesting and relevant to a person’s daily life than most other sciences. As we shall see in this commentary, neuroscience opportunities are increasing for teenagers outside the standard curriculum, although they are slow to develop inside the curriculum (Myslinski, 2001).

## Neuroscience for Neuroscientists

The size and diversity of neuroscience as a separate biomedical entity has increased ever since the mid-20th century. The International Brain Research Organization (IBRO) followed by the Society for Neuroscience (SfN) gave structure and identity to neuroscience as a separate field. President George H. W. Bush declared the 1990s as the Decade of the Brain, and as a result, funding for the National Institutes of Health doubled. Subsequently, other organizations, university departments, and academic journals focused on neuroscience were created, but these were mainly in the realm of biomedical professional schools. Neuroscience then became more visible at the undergraduate level with the advent of courses and degrees in neuroscience. As a result, the Faculty for Undergraduate Neuroscience (FUN) was created. This growth led to the creation of The Association of Neuroscience Programs and Departments, which gave strength in numbers for fund raising, political influence, publications, and public relations.

## Neuroscience for Everyone

Neuroscience is not just for neuroscientists; it is for everyone. Topics such as child development, memory, pain, sleep, fear, music, intellect, vision, hearing, addictions, language, movement, learning, neuroethics, and aging can be applied to the daily lives of teachers, parents, politicians, journalists, film makers, advertisers, web designers, etc. (Myslinski, 2007a). Understanding neuroscience can help the lay public understand the >1000 disorders of the brain and nervous system, including the side effects of other disorders, such as COVID-19. Scientists must communicate with the general public to gain their support, to improve their quality of life, and to make them aware of advances in the field. In the 1990s, the Dana Alliance for Brain Initiatives created Brain Awareness Week (BAW) for that purpose. Since its founding in 1996, BAW has included the participation of >7300 partners in 120 countries. Activities include exhibits, visitations, brain fairs, lab tours, film festivals, and programs such as “Brains Rule!” (Zardetto-Smith, 2003). These activities use limbic learning (emotion-enhanced learning) to get and retain the attention of the lay public (Myslinski, 2007a). Outdoor brain games, such as Cochlear Hopscotch or Synaptic Tag, brain songs, such as the Dendritic Song or Brain Rap, indoor brain games, such as Brain Bingo or Neuro-jeopardy, and even brain jokes have the ability to produce excitement and laughter while learning about the brain. Even constructive fear can help learning when it comes to drug abuse and wearing safety helmets. Sitting and reading about the brain may give you and me a thrill, but it is not very memorable for the average lay person. However, if they are exposed to the same information while singing a brain song, winning a neuroscience competition, playing a brain game, or crying during a movie about a child with a brain disorder, they will have a learning experience that will last a lifetime. Portugal transformed a local shopping center into a BAW performance space. Nigeria broadcasted BAW programing on the role of the brain in learning and leadership to more than one million radio listeners. Argentina constructed a giant structure representing the brain in the center of Cordoba City. Inside the brain structure there were a number of different activities, such as drawing pictures of the brain, attending short public lectures, and enjoying a brain-themed music and dance show. Turkey conducted laboratory visits, while India conducted a brain trivia initiative, complete with an orchestra performance. Israel featured a series of brain-related film screenings. In Grenada, WI, the effective collaboration of the K–12 education system, St. George’s University, the government and the Church provides neuroscience education to their eager students (Myslinski, 2018) In Brazil, high school and postgraduate students worked together to create robots that demonstrate sensorimotor integration. Whereas popular media in books, cinema and the Internet have occasionally portrayed science and scientists in a negative light, they have also excited, inspired, and motivated future neuroscientists (Zehr, 2016). Advocates, such as Paul Aravich, are vocal and passionate about engaging local and national communities and governments about the importance of the human brain and neuroethics (Shields, 2003; Myslinski, 2007a). Popular authors such as Harold Klawans (Klawans, 2000), Oliver Sacks (Sacks, 1974), and Michio Kaku (Kaku, 2014) translate the complexities of the brain and mind into language that is easily understood. Sacks’ book, *Awakenings*, was made into a 1990 Oscar-nominated film starring Robin Williams and Robert De Niro. Additionally, celebrities, such as Christopher Reeve and Michael J. Fox, have also served as spokesmen for the public awareness of neuroscience and research.

## Neuroscience for Teenagers

Despite the top-down evolution of neuroscience awareness, high schools have been resistant to integrating it into their curricula. As a result, extracurricular clubs and organizations have grown up to fill the gap, including such international organizations as The International Brain Bee (IBB), and the International Youth Neuroscience Association (IYNA; Myslinski, 2019a).

## The IBB

The IBB is the preeminent global neuroscience competition for teenage students (https://thebrainbee.org). Worldwide, there are ∼local chapter competitions in ∼50 countries on six continents. The chapter winners then compete in their respective National Championships to earn the right to compete in the World Championship. They are tested on their knowledge of the human brain with oral and written tests including a neuroanatomy examination using human brains, and a patient diagnosis component using actors. Students study online resources that are free to download and have been translated into multiple languages, such as *Brain Facts and Neuroscience: The Science of the Brain*. Past venues for the World Championship include Baltimore, Toronto, Montreal, Toronto, San Diego, Florence, Cape Town, Vienna, Washington, Cairns, Copenhagen, Berlin, and Daegu. Past hosts of the World Championship include the World Congress of Psychology, the World Congress of Neurology, the International Society for Neurochemistry, the Canadian Neuroscience Society, the University of Maryland (Baltimore), the Federation of European Neuroscience Societies (FENS), the American Psychological Association (APA), and IBRO. Local coordinators are neuroscientists, neurologists, teachers and administrators from high schools, museums, and industry who are interested in science education and community outreach. Sponsors include colleges, universities, foundations, museums, institutes, societies, and commercial companies and businesses. Recently, the IBB has been incorporated as a nonprofit organization. Six neuroscience organizations are taking a leadership role and investing their energy, time, resources and funds to make the IBB better than ever, now and into the future. They are the Dana Foundation, IBRO, SfN, FENS, APA, and the Alzheimer’s Association. The IBB’s purpose is to motivate young men and women to learn about the human brain, and to apply that knowledge to their daily lives; as well as to inspire them to enter careers in the basic and clinical brain sciences to help treat and find cures for brain disorders. An estimated 50,000 students compete annually. More than 100 newspapers, radio, television stations and web sites cover the IBB. Presidents, ambassadors and other public officials have recognized the IBB. The IBB has given high school students the excitement of seeing a real human brain, of being applauded by thousands of neuroscientists, and winning neuroscience competitions. Many former competitors are now working in neuroscience, neurology, psychology and related fields. The 2001 World Brain Bee Champion, Arjun Bharioke, is now a Postdoctoral Fellow working on neural circuits at the University of Basel in Switzerland. The 2008 World Champion, Elena Perry, graduated from Yale University and is now a Postdoctoral Research Fellow at Genentech in the San Francisco Bay Area. The 2017 World Champion, Sojas Wagle, is a student at Brown University studying Psychiatric Epidemiology. The 2019 World Champion, Yidou (Gwen) Weng, is now a student at National University of Singapore studying Computational Biology. Some Brain Bee Alumni go into neuroscience-related but nonlaboratory careers. The 2002 World Third Place Winner, Julianne McCall, is now working on Science Policy in the California Governor’s Office of Planning and Research (Sukel, 2022; https://online.flippingbook.com/view/40003043/44/).

The Brain Bee motto is: Building Better Brains to Fight Brain Disorders. Here are some comments of former Brain Bee competitors:

Australia: It changed the way I view the world and myself.

Canada: The Brain Bee opened my eyes and deepened my passion & understanding for neuroscience.

Egypt: I’m in love with every single aspect of the IBB.

Germany: It really motivated me towards neuroscientific research, and today I can’t really imagine ever doing anything else.

Grenada: I found the Brain Bee Program to be one of great hospitality and elegant organization. It was certainly an influential one that widened my view of the neuroscience field and the surplus of possibilities that it holds for the future of humanity.

Hong Kong: It sparked my interest in the brain, gave me an amazing experience that I will never forget, and made me fall in love with neuroscience.

Iran: It is the best experience I’ve ever had in my life.

Italy: It was with no doubts the best experience I’ve done so far in my whole life. Absolutely!

Kenya: It has formed a good basis for my future, helped raise my awareness about brain disorders, and enabled me to interact with people from different cultures.

New Zealand: I honestly view my participation as a dividing line in my life - there is before Brain Bee and there is after Brain Bee.

United States: The Brain Bee was a terrific way to learn about a fascinating discipline of science that is rarely taught in the traditional science class. I have always been interested in neuroscience but have never had an opportunity to actually learn in depth until the Brain Bee.

## The IYNA

The IYNA is a global, 501(c)(3) nonprofit completely run by high school students dedicated to inspiring the next generation of neuroscientists (Myslinski, 2018). Its mission is to introduce students to the excitement of the brain. It serves as a network uniting neuroscience clubs and interested individuals for mutual benefit. IYNA pursues this goal through a variety of means, including the development of a high school level neuroscience curriculum, promoting the founding of IYNA Chapters around the world, and fostering communication between budding neuroscientists. One of their projects is the Modern Youth Education, Leadership, and Inquiry in Neuroscience (MYELIN) initiative, which seeks to develop a high school-level neuroscience curriculum. They hope to develop a classroom-ready course which will introduce students around the world to the wonders of the brain. It will include learning objectives, lecture slides, supplementary worksheets with questions and answers, lab instructions, and a teacher’s guide. When finished, MYELIN will be available for free to all teachers and students. Another project of IYNA is the *IYNA Journal*. It is a bimonthly publication written, edited, and published entirely by student members of the IYNA. The journal not only encourages student writers to develop their scientific comprehension and communication skills, but also provides an excellent educational resource to readers. IYNA has 560 registered chapters with >8000 members representing 125 countries (https://youthneuro.org).

## Neuroscience for the Future

The human brain makes us who we are. To understand ourselves we have to understand our brains. Neuroscience is for everybody, all ages, all around the world. It is never too early to involve neuroscience in our lives, or too late. The future will see the growth of neuroscience awareness from high schools to elementary schools and even preschools (Myslinski, 2019b, 2020). Already, there are web sites focused on increasing awareness, such as “Neuroscience for Kids.” There is now a program in the Israel Sci-Tech School, in collaboration with Tel Aviv University, that provides up to 120 h of neuroscience instruction to middle and high school students. Neuroscience will become a greater part of senior citizen centers and the lives of the elderly. In the very far future our brains, and the function of our brains, our minds, will evolve to adapt to changes on earth and beyond earth (Kaku, 2014). As we integrate with artificial intelligence, our concept of “self” will change. We must include the brain and mind in our concept of what it means to be human. It is more important than ever to provide our youth with a strong neuroscience foundation (Myslinski, 2000b), and it is not too early to start.[Fig F1][Fig F2]

**Figure 1. F1:**
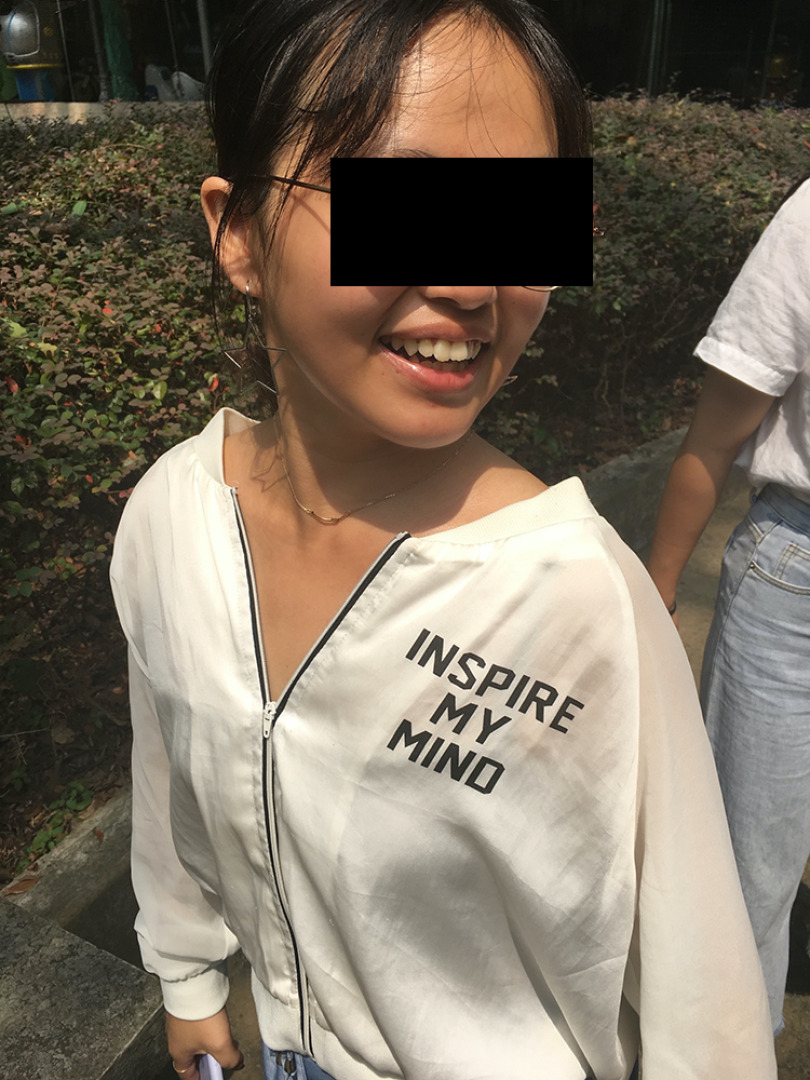
An inspired student in China. Photograph taken during the author’s speaking tour of Brain Bee Chapters in China and South Korea.

**Figure 2. F2:**
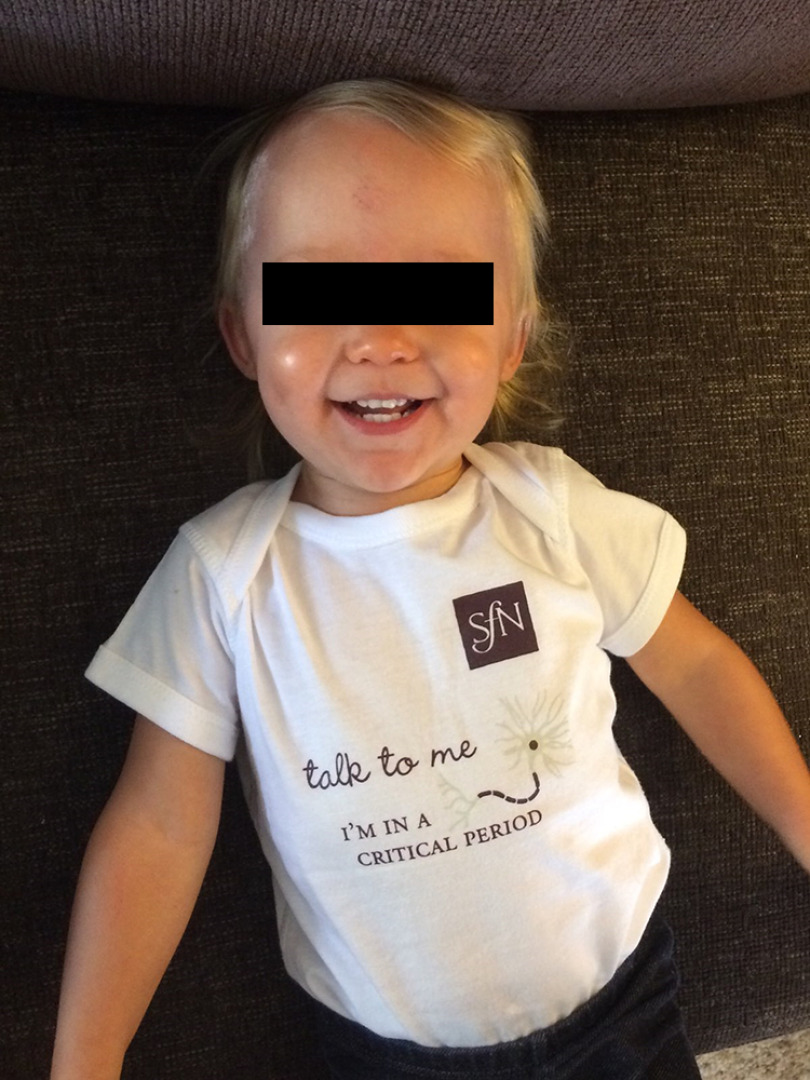
Fifty percent of our brains is hard-wired at birth. The concept of neuroplasticity tells us that the rest is waiting for us to tell it what to do. Our experiences, sensations, thoughts, and movements tell our brains what is important and how to develop. Not only do our brains create our behavior, but our behavior can create our brains.

## Synthesis

Reviewing Editor: Christophe Bernard, INSERM & Institut de Neurosciences des Systèmes Decisions are customarily a result of the Reviewing Editor and the peer reviewers coming together and discussing their recommendations until a consensus is reached. When revisions are invited, a fact-based synthesis statement explaining their decision and outlining what is needed to prepare a revision will be listed below. The following reviewer(s) agreed to reveal their identity: NONE.

This is a nice and timely piece.

I have a few comments

1) It is a bit US-centric; the readership of both SfN journals is international. I don’t know how other countries fare in terms of neuroscience in high school. But in France there is is a program that teaches the basics during the last year: http://acces.ens-lyon.fr/acces/thematiques/neurosciences/ressources-pedagogiques/terminale-s-programme-applicables-des-la-rentree-2020

This site shows you what the teachers need to know to teach.

It would be interesting to check what is done in the UK, Germany, Israel (I know that they start to teach neuroscience very early) etc.

2) It may be interesting to discuss how it would be possible to implement neuroscience in US/Canada high schools - perhaps building on what other countries are doing. That is to say to give more perspective? btw, are the winners of the competitions exposed to neuroscience in high school in their countries?

3) It would be nice to have active links when you refer to things available of the web.

4) I am not sure what the testimonies bring about, they may not be necessary. You could use the picture of the girl for a graphical abstract (or that of your(?) baby).

Minor

Please reduce the number of citations to 10 max.

Please define STEM

I know Robert but not Peter de Niro :-)
